# Adherence to multiple micronutrient powder among young children in rural Bangladesh: a cross-sectional study

**DOI:** 10.1186/s12889-015-1752-z

**Published:** 2015-04-30

**Authors:** Mirak Raj Angdembe, Nuzhat Choudhury, Mohammad Raisul Haque, Tahmeed Ahmed

**Affiliations:** Department of Public Health, Central Institute of Science and Technology, Kathmandu, Pokhara University, Pokhara, Nepal; James P. Grant School of Public Health, BRAC University, Dhaka, Bangladesh; Centre for Nutrition and Food Security, International Centre for Diarrhoeal Disease Research, Bangladesh (icddr, b), Dhaka, Bangladesh; BRAC Health Programme, BRAC, Dhaka, Bangladesh

**Keywords:** Multiple micronutrient powder, Adherence, Young children, Bangladesh

## Abstract

**Background:**

Multiple micronutrient powder (MMNP) can be sprinkled onto any semisolid food and can be given to young children to address iron deficiency anemia. The female community health volunteers of BRAC (an NGO) known as *Shasthya Shebikas* (SS) sell MMNP sachets during their regular household visits. Currently there are no data on adherence or real uptake of MMNP by children. The objective of the study was to assess adherence to MMNP and associated factors among children aged 6–59 months in rural Bangladesh.

**Methods:**

A cross sectional study was conducted in Saturia Sub-district among 78 children aged 6–59 months who were fed MMNP supplied by BRAC SS in the past 60 days. A one stage cluster sampling technique was used to select mothers with eligible children. Semi-structured questionnaire was used for interviews. A logistic regression model was developed to obtain adjusted odds ratios (AOR) with 95% CI.

**Results:**

Sample mean adherence was calculated to be 70%. In multivariate analysis, age of mother in years (AOR = 0.74, 95% CI: 0.61-0.88), households belonging to poorer (AOR = 0.01, 95% CI: 0.00-0.68), middle (AOR = 0.04, 95% CI: 0.00-0.35) and richer (AOR = 0.11, 95% CI: 0.01-0.84) wealth quintiles and mothers who prefer to feed flexibly (AOR = 0.03, 95% CI: 0.00-0.26) were significantly associated with high adherence. Further, for every one unit increase in visit by BRAC SS in the past 60 days, the odds of having high adherence significantly increased by 55% (AOR = 1.55, 95% CI: 1.09-2.20).

**Conclusions:**

SS are the key to improving adherence through regular visits to households of MMNP users. However, expanding coverage beyond the vicinity of the SS’s household is a challenge. Perception of families whose children have low adherence should be studied.

## Background

It is generally assumed that 50% of anemia cases are due to iron deficiency [1] and these are further exacerbated by infectious diseases in resource poor settings [2]. In young children impaired cognitive, motor and behavioral development are the major health consequences of iron deficiency anemia (IDA) [3-5]. Iron deficiency can be addressed principally through three approaches: first is dietary diversification which is about including foods rich in absorbable iron; second is fortification of staple food items such as wheat flour; and third is the provision of iron supplements which acts as an alternative strategy when the first two are not feasible in terms of available resources [6].

Existing literature has highlighted supplementation with iron syrup/drops or tablet being prone to poor adherence because of several factors that include difficulty swallowing tablets [7], drops having gastrointestinal side effects like abdominal discomfort, unpleasant and strong metallic taste, staining of a child’s teeth if not wiped off immediately and complicated dosing instructions [8,9]. Limited shelf life inherent to liquid preparations and expensive transportation costs owing to the weight of bottles were further disadvantages [10].

In order to alleviate these problems multiple micronutrient powder (MMNP) was developed which is available as single dose sachet of dry powder containing lipid-encapsulated iron and other micronutrients that can be sprinkled onto any semisolid food [6] and given to young children. The lipid-encapsulation coating prevents iron from dissolving into the food and therefore prevents any change in color, flavor or taste [6]. This is the concept of home fortification.

In Bangladesh, the anemia prevalence (hemoglobin concentration <110 g/L) in children aged 6–59 months was 68% in rural areas [11]. After doing a series of studies on MMNP [12-14], BRAC (a local NGO formerly known as Bangladesh Rural Advancement Committee) since December 2009 has been operating MMNP program called “Bangladesh Sprinkles Program” in 61 districts of Bangladesh to decrease the prevalence of IDA amongst children aged 6–59 months. The MMNP used in this program is locally marketed as *Pushtikona* and has 15 ingredients. A single sachet, 1gm dry powder of *Pushtikona* contains: Vitamin A: 0.4 mg, Vitamin C:30 mg, Vitamin D:0.005 mg, Vitamin E:5 mg, Vitamin B1:0.5 mg, Vitamin B2:0.5 mg, Niacin:6 mg, Pyridoxine:0.5 mg, Vitamin B12:0.0009 mg, Folic Acid:0.15 mg, Iron:10 mg, Zinc:4.1 mg, Copper:0.56 mg, Selenium: 0.017 mg & Iodine:0.09 mg.

The female community health volunteers of BRAC known as *Shasthya Shebikas* (SS) are trained to provide health services including selling of essential health commodities such as MMNP. Currently, there are about 80,000 SS all over Bangladesh [15]. They buy each box (containing 30 sachets) of MMNP at a price of 48 Bangladeshi Taka/BDT or 0.6 USD (1 USD = 80.1 BDT as of December 24, 2012) and then sell it to the mothers at a price of 75 BDT or 0.9 USD (2.5 BDT or 0.03 USD per sachet) during their regular household visits. The price difference acts as their incentive.

One sachet is given to a child every day for 60 days followed by a period of 120 days without MMNP supplementation before it is started once again. It is repeated until the child turns five. This schedule has been implemented since July 2012. In the previous schedule, child had to be fed every alternate day. The SS also provide instructions to the mothers on use of MMNP together with behavior change communication (BCC) messages. Each SS is assigned a letter grade ranging from A to D (A being best) based on their performance by the BRAC office. Their performance is assessed in terms of the number of products they sell.

Over the last decade the efficacy and safety of home fortification with MMNP has been well established [16-20]. A recent review of 16 randomized control trials estimated significant improvement in hemoglobin concentration and reduction of IDA by 57% [21]. However, from an implementation perspective adherence and acceptability are other significant issues of concern which will determine the effectiveness of such large scale interventions. The current major challenge relates to unavailable data on adherence or real uptake of MMNP by children in Bangladesh. High levels of adherence are necessary for MMNPs to improve health but little is known about the relative importance of various factors influencing children’s adherence to these interventions. This study aimed to assess adherence to MMNP and associated factors among children aged 6–59 months in rural Bangladesh. The study will provide the necessary information to design and implement strategies that might be helpful for improvement of such programs and eventually contribute in judging the effectiveness of the program.

## Methods

### Ethics statement

The study protocol was approved by the ethical review committee at James P Grant School of Public health, BRAC University, Dhaka. Prior to interview, written informed consent was obtained from the mothers who consented on behalf of their children. In cases where the mother was illiterate, verbal consent was taken from the mother following a detailed description of the study protocol and written consent was taken from her husband who consented in writing on behalf of both the mother and the child. The interviewer also signed a statement for verifying that he had provided the necessary information written in the informed consent form and that each respondent had consented to participate in the study. All data were coded to remove identifying information and secure confidentiality. Respondents were not compensated for participation in the study.

A cross sectional study was carried out in Saturia *Upazilla* (Sub district) of Manikganj District in rural Bangladesh among children aged 6–59 months who were fed with MMNP supplied by BRAC SS in the past 60 days. It is one of the 61 BRAC MMNP program districts where Alive and Thrive program is also operational. Alive and Thrive is operational in 16 districts since 2009 and aims to reduce malnutrition and death caused by poor infant and young child feeding practices. It is one of the platforms for MMNP service delivery.

**Figure 1:**
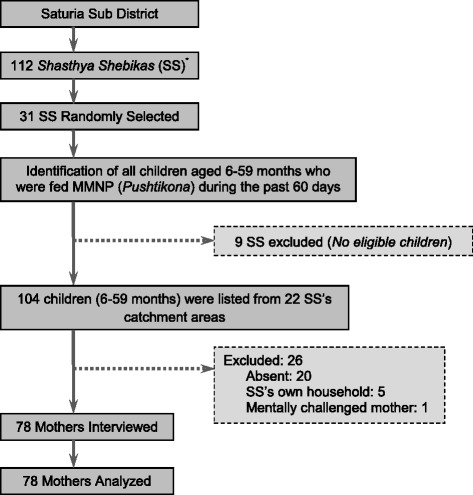
Diagram of selection of *shasthya shebikas* (*female community health volunteers) and eligible children in the study.

According to previous study [13] conducted in 9 *Upazillas* of Bangladesh, the overall adherence was 81% in children aged 6–23 months. With a 95% confidence level and 10% margin of error, sample size was 59. A design effect of 1.2 was added to minimize the clustering effect as suggested in National Nutrition Programme Baseline Survey 2004 [22]. Taking into account a non-response of 10%, final sample size was calculated to be 78. A one stage cluster sampling technique was used where each SS was technically a cluster. There were 112 SS working in Saturia. A list of all the SS working in the *Upazilla* was obtained from the local BRAC office. Simple random sampling was used to select 31 SS from the list. In each of the SS’s catchment areas all children aged 6–59 months who were fed MNP during the past 60 days were identified. Nine out of 31 selected SS did not have any eligible children and were excluded. A total of 104 eligible children were listed from 22 SS’s catchment areas. Out of these, mothers of 20 eligible children were absent during interview and were not followed up thereafter. Five of the children were from the SS’s own household and one of the mothers was mentally challenged and unable to answer interview questions. Thus after excluding 26 children overall we ended up interviewing and analyzing data from 78 mothers (Figure 1).

Data collection was carried out in November 2012 by trained interviewers using a standardized pre tested semi-structured questionnaire which was drafted in English and translated in to Bangla for field implementation. The questionnaire inquired information on socio-demographic characteristics, morbidity status, childhood anemia and MMNP related knowledge; mothers experience with MMNP and BRAC MMNP program; and adherence to MMNP. The second part of the questionnaire measured acceptability. Those mothers who were currently feeding MMNP were asked to show the unused sachets of MMNP in order to determine the number of sachets consumed out of those that were bought the last time. The date of purchase was used to calculate the days taken for consumption. This information was used to calculate adherence.

The outcome variable adherence was calculated by dividing the number of sachets consumed by days taken to consume the number of sachets. For further analysis, the outcome variable was dichotomized based on mean value such that *0 = Low adherence* (≤ mean) and *1 = High adherence* (> mean). Illness in the past 15 days was measured through 13 dichotomous questions^a^ on signs of illness followed by a question that inquired whether a dose/sachet of MMNP was missed because of illness.

### Wealth index

It was constructed using household asset data [23]. It comprised of ownership of several consumer items: electricity, wardrobe, table, chair/bench, dining table, bed, radio/2-in-1, television, cycle, motorcycle, sewing machine, electric fan and telephone/mobile. Principal component analysis was used to generate and assign weight (factor score) to each asset. Weights were assigned based on the first component which accounted for 30% of total variance. The asset scores for individual household were then computed and divided into quintiles from one (poorest) to five (richest).

### Childhood anemia related knowledge

It was measured through 5 questions on causes, signs, consequences and prevention/treatment. Each correct answer to these questions was scored one else zero. Thus the total score ranged from 0 to 5. The scores were then converted to a 0–100 scale with higher scores indicating better knowledge. The final constructed scale had a Cronbach’s alpha reliability coefficient of 0.89.

### MMNP related knowledge

It was initially measured through 9 questions on product information, dosage and usage. However after preliminary analysis the Cronbach’s alpha reliability coefficient of the scale with these 9 variables was unacceptable. Principle component analysis was performed to determine the variables that were to be retained in the final scale. Consequently, 7 out of 9 items were excluded and for final analysis, scale was constructed with 3 items. These included questions on reasons, frequency and quantity of MNP use. Each correct answer to these questions was scored one else zero. Thus the total score ranged from 0 to 3. The scores were then converted to a 0–100 scale with higher scores indicating better knowledge. The final constructed scale had a Cronbach’s alpha reliability coefficient of 0.65.

### Acceptability

It was measured through 8 questions in 2 dimensions or subscales including ease of use (3 items) and organoleptic properties (5 items) [24]. Ease of use measured acceptability of packaging, preparation and storage. Organoleptic properties measured acceptability of flavor, odor, color, texture and overall liking of the food to the child after being mixed with MMNP. The responses to these questions were measured on a 7 point hedonic scale ranging from 1 to 7 (1 = dislike extremely 2 = dislike moderately 3 = dislike slightly 4 = neither dislike nor like 5 = like slightly 6 = like moderately 7 = like extremely) where higher values indicated greater acceptability. Summary scores were calculated for each dimension separately and then converted to a 0–100 scale with higher scores indicating higher acceptability. The final constructed scale had a Cronbach’s alpha reliability coefficient of 0.87 for ease of use and 0.96 for organoleptic properties.

### Covariates

Couple of factors was considered as covariates or potential confounding variables. First, in sampling all SS may not have identical level of performance and eligible children. It was adjusted by including the variable on SS grade. Each SS is assigned a letter grade ranging from A to D (A being best) based on their performance by the BRAC office. Their performance is assessed in terms of the number of products they sell. We collected information on grades that had been revised recently (a month before data collection) from the BRAC office and entered it as a variable that was adjusted for when developing the multivariate model. Second, there were children who skipped a sachet of MMNP because of any illness in past 60 days. The SS instruct the mothers to skip sachets when the child is ill. Such children were considered adherent for that period and calculations were adjusted accordingly.

Data were entered in SPSS (ver.17) and analyzed with Stata (ver.12) using ‘Svy’ commands for survey data analysis. It takes survey design characteristics into account and adjusts calculations accordingly. Appropriate sample weights were also used to adjust for the cluster sampling design. The Chi-square test and Fisher’s exact test were used to determine statistically significant differences for categorical variables and Mann Whitney test for quantitative variables. A logistic regression model was developed to examine the effects of predictor/independent variables on adherence. Variables were fitted in the model in blocks: *1.* Socio-demographic *2.* Morbidity related *3.* Mother’s perception related *4.* Program related *5.* Knowledge related *6.* Acceptability related *7.* SS grade variable. At each stage, the least significant variable was excluded until the model contained only statistically significant factors and the covariates. Multicollinearity was assessed. Adjusted odds ratios (AOR) were calculated with 95% confidence intervals (95% CI). A two tailed *P*-value of <0.05 was considered to be statistically significant.

## Results

The background characteristics of respondents are presented in Table 1. The mean age of children was 25 months and almost half (47%) were females and 53% were males. On average every household had 2 children. The birth order of reference child in most (82%) cases was either 1 or 2. A large percentage of mothers were currently married (99%), secondary level educated (46%) and housewives (95%). Those other than housewives were engaged in small business, service or were school teachers (Data not presented in Table). The average household size was 6 members. About 36% of households had sanitary latrine (water sealed with septic tank) while others had slab latrine (60%), pit latrine (1%) and hanging latrine (3%) (Data not presented in Table).

**Table 1:** **Socio-demographic characteristics of the respondents**

**Variables (N = 78)**	**Mean or Percent (as appropriate)**
Age of the child in months (± SD)	24.9 ± 14.3
Sex of the child, Female (n)	47.4 (37)
Number of live children (± SD)	1.8 ± 0.8
Birth order of reference child (n)	
1	41.0 (32)
2	41.0 (32)
≥3	18.0 (14)
Age of the mother in years (± SD)	25.2 ± 3.9
Marital status, Currently married (n)	98.7 (77)
Mother’s education (n)	
No education	21.8 (17)
Primary (1–5)	26.9 (21)
Secondary (6–10)	46.2 (36)
Higher	5.1 (4)
Mother’s occupation, Housewife (n)	94.9 (74)
Household size (± SD)	5.6 ± 2.1
Type of latrine, Sanitary (n)	35.9 (28)
Wealth index quintile (n)	
Poorest	20.5 (16)
Poorer	20.5 (16)
Middle	19.2 (15)
Richer	24.4 (19)
Richest	15.4 (12)

### Morbidity status

A large percentage (88%) of mothers reported that their child showed at least one sign of illness in the past 15 days (Table 2). The top three signs of illness were fever (49%), vomiting (31%) and itching in the body or head (26%). Likewise, nausea (18%), diarrhea (13%) and ARI related illness (10%) were also reported. Around 19% of the mothers reported that they skipped a sachet of MMNP because of any illness (child) in the past 60 days. Nearly half (46.7%) of those who skipped a sachet had fever in the past 15 days (Data not presented in Table).

**Table 2:** **Morbidity status of child**

**Variables (N = 78)**	**% (n)**
Children showing any one sign of Illness in the last 15 days	88.5 (69)
Fever	48.7 (38)
Vomiting	30.8 (24)
Itching in the body or head	25.6 (20)
Nausea	17.9 (14)
Diarrhea	12.8 (10)
ARI related illness	10.3 (8)
Skipped a sachet of MMNP because of any illness in past 60 days	19.2 (15)

### Adherence to MMNP

**Figure 2:**
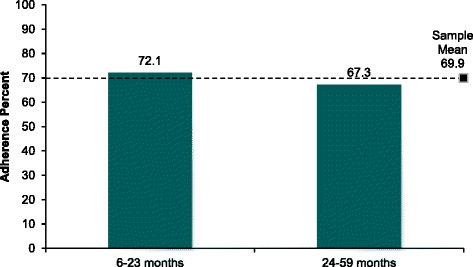
Adherence to MMNP by age group of child (in months).

The sample mean adherence was calculated to be around 70% as illustrated by the horizontal reference line in Figure 2. The same figure also shows the mean adherence in two age groups of children. Among children aged 6–23 months, the mean adherence was 72%. On the other hand, children aged 24–59 months had a mean adherence of 67%. The mean adherence was highest among the poorest (78.4%), followed by the richest (76.6%), richer (70.9%), middle (70.3%) and poorer (54.9%) (Data not presented in Figure). For further calculations, adherence was dichotomized based on mean value such that adherence of ≤ 70% was considered as low adherence and adherence of >70% was considered high adherence.

None of the mothers reported any barriers to using MMNP*.* Of all mothers, 1% shared MMNP sachets with other household members, 3% reported that some of the sachets were spoilt or lost and 4% reported that they shared the MMNP mixed semi-solid food with someone besides the reference child (Data not presented in Figure).

### Mothers' experience with MMNP

More than half (60%) of the mothers had perceived positive health gains in their children after they started giving MMNP (Table 3). These health gains included general improvement in health, increase in weight, increase in strength, increase in digestion and improved nutrition. Additionally, 36% of the mothers perceived an increase in appetite, 17% reported that it prevented diseases and 8% reported mental development. On the contrary only 1.3% of mothers reported a negative effect which was vomiting. About 19% of the mothers did not observe any changes in their children. Nearly half (46%) of the mothers perceived changes in food after mixing MMNP*.* Of those who perceived changes majority (92%) said the change was in color. Others (8%) said the change was in smell and texture.

**Table 3:** **Mothers’ experience with MMNP**

**Variables (N = 78)**	**% (n)**
Perceived changes in child’s health after feeding MMNP*	
Positive health gains	60.2 (47)
Increased appetite	35.9 (28)
Prevented diseases	16.7 (13)
Mental development	7.7 (6)
Vomiting	1.3 (1)
No changes observed	19.2 (15)
Perceived changes in food after mixing MMNP	46.2 (36)
Perceived changes in color (n = 36)	91.7 (33)
Perceived other changes (n = 36)	8.3 (3)

*multiple response.

### Mothers' experience with BRAC MMNP program and activities

Majority (74%) of the mothers preferred the current daily feeding schedule of MMNP compared to previous schedule when feeding was every alternate day. During the past 60 days BRAC SS visited the households 7 times on average. A large percentage (96%) of respondents reported that SS provided information on anemia and/or MMNP during their last visit. Approximately 15% of the mothers had watched or heard or read about MMNP from sources other than the BRAC SS. (Table 4)

**Table 4:** **Mothers' experience with BRAC MMNP program and activities**

**Variables (N = 78)**	**Mean or Percent (as appropriate)**
Schedule preference (n = 74)	
Feeding Daily	74.3 (55)
Feeding Flexibly	25.7 (19)
Visits by BRAC SS in the past 60 days (± SD)	6.6 ± 4.3
SS talked about anemia and/or MMNP during last visit (n)	96.1 (75)
Watched/heard/read MMNP related information (n)	15.4 (12)

### Mothers' knowledge and acceptability

The mothers had a mean childhood anemia knowledge score of 54.6 ± 38.9 and a mean MMNP knowledge score of 95.7 ± 15.5. Acceptability scores were calculated for two dimensions. First, the mean ease of use summary score was 91.6 ± 10.4. It was calculated based on 3 items: the median ratings for packaging and preparation were 7 (4, 7) -“Liked extremely” and for storage the median rating was 7 (5, 7) -“Liked extremely”. Second, the mean organoleptic properties summary score was 75.7 ± 23.6. It was calculated based on 5 items with median ratings of 6 (1, 7) - “Liked moderately” for color, flavor, texture, smell and overall likeability.

### Factors associated with adherence to MMNP

Table 5 shows the bivariate analysis between the outcome variable adherence and other factors. As many variables were analyzed, only those that were significant and/or included in the final multivariate model are presented. In bivariate analysis statistically significant (*P* < 0.05) differences were observed between high and low adherence group with respect to schedule preference (*P* = 0.007) and mean number of visits by BRAC SS in the past 60 days (*P* = 0.014).

**Table 5:** **Factors associated with adherence to MMNP**

**Variables (N = 78)**	**High Adherence (n = 39)**	***P*** **-value**	AOR* (95% CI)	***P*** **-value**
	**% (n)**			
**SOCIO-DEMOGRAPHIC FACTORS**				
Mean age of the mother in years (± SD)	24.3 ± 3.1	0.088^a^	0.74 (0.61-0.88)	0.002
Wealth index quintile				
Poorest	62.5 (10)	0.171^b^	1.00	
Poorer	25.0 (4)		0.01 (0.00-0.68)	0.033
Middle	46.7 (7)		0.04 (0.00-0.35)	0.006
Richer	52.6 (10)		0.11 (0.01-0.84)	0.035
Richest	66.7 (8)		0.93 (0.09-10.23)	0.953
**PROGRAM RELATED FACTORS**				
Schedule preference (n = 74)				
Feeding Daily	60.0 (33)	0.007^b^	1.00	
Feeding Flexibly	21.1 (4)		0.03 (0.00-0.26)	0.003
Mean visits by BRAC SS in the past 60 days (± SD)	7.7 ± 4.9	0.014^a^	1.55 (1.09-2.20)	0.018
**SS** ^**c**^ **Grade**				
Grade A	42.2 (19)	0.409^b^	1.00	
Grade B	56.3 (9)		0.26 (0.01-6.90)	0.405
Grade C	60.0 (6)		4.70 (0.69-32.06)	0.108
Grade D	71.4 (5)		0.11 (0.00-3.22)	0.188
**MORBIDITY RELATED FACTOR**				
Skipped a sachet of MNP because of any illness (child) in past 60 days				
No	55.6 (35)	0.083^b^	1.00	
Yes	26.7 (4)		0.10 (0.00-2.57)	0.154

Covariates adjusted were mother’s age, wealth index, schedule preference, number of visits by BRAC SS in the past 60 days, skipped a sachet of MMNP because of any illness (child) in past 60 days and SS grade. Only those that were significant in logistic regression are presented.

^*^Adjusted Odds Ratio.

^a^Mann–Whitney test (2-tailed).

^b^Fisher’s exact test.

^c^ *Shasthya Shebika (female community health volunteers).*

In multivariate analysis, age of the mother in years (AOR = 0.74, 95% CI: 0.61-0.88), households belonging to the poorer (AOR = 0.01, 95% CI: 0.00-0.68), middle (AOR = 0.04, 95% CI: 0.00-0.35) and richer (AOR = 0.11, 95% CI: 0.01-0.84) wealth quintiles and mothers who prefer to feed flexibly (AOR = 0.03, 95% CI: 0.00-0.26) were significantly associated with having high adherence. Further, for every one unit increase in visit by BRAC SS in the past 60 days, the odds of having high adherence significantly increased by 55% (AOR = 1.55, 95% CI: 1.09-2.20) (Table 5).

## Discussion

Iron supplements have long been associated with poor adherence, a factor which led to the development of MMNP. Interventions like MMNP that are targeted to reduce anemia prevalence in rural communities will work only if the levels of adherence are high. So far adherence has been measured only in trials which have found levels to be variable and in some instances only as good as drops and syrups [20]. This was a cross-sectional study to measure adherence at community level within a large scale program setup that covers most of the country. The study found an adherence of 70% among children aged 6–59 months in a rural *Upazilla* in Bangladesh. For a similar daily 2 months schedule, previous studies in Bangladesh have found an adherence of 81-100% [12-14,25]. Most of these studies were trials which are conducted in controlled settings where field workers deliver and monitor intervention on a regular basis. This may be a possible explanation for relatively much higher adherence reported by these studies.

Children whose mothers came in frequent contact with SS were significantly more likely to have high adherence than others. In fact most of the MMNP users were located nearer to the SS’s household and sometimes forming a cluster around the SS’s household. Further, SS’s skills and knowledge might have affected the mother’s level of knowledge*.* Unlike other studies on pregnant women [26] knowledge scores did not differ significantly between high and low adherence groups. The mean knowledge score for MMNP (95.7 ± 15.5) was higher than that for anemia (54.6 ± 38.9). This might indicate that most mothers were simply following instructions on MMNP use without having the understanding of why it is used. It seems mothers do not make knowledge based decisions, rather follow instructions that are reinforced by the presence of SS in their household. Other studies have reported similar results where the advice of a health worker, in this case midwife positively affected adherence to iron supplementation among women of reproductive age [27] and among pregnant women [28]. Likewise, a study from Mali concluded quality of counseling to be vital for adherence among pregnant women. They have emphasized ‘minimum, consistent, and easily understandable information and counseling’ [29]. The effectiveness of community health workers has also been evaluated in other community based interventions where they contributed to reduction of perinatal and neonatal mortality in rural Pakistan [30]; and reduction of anemia prevalence by increasing coverage of pregnant women attending antenatal care in Thailand [31].

In our study wealth index is a relative estimate within the group who can actually buy MMNP. Households categorized as poor or rich may not be truly poor or rich. Nevertheless we found that poorest were significantly more likely to have high adherence. It might be because economically vulnerable groups are more receptive to health messages and instructions. Alternatively, the prosperous ones are insensitive to instructions coming from a SS who may not be of the same social or economic class. Similar behavior has been observed with exclusive breast feeding which is also practiced more among the poor than better off [32]. Further, cost was not specifically mentioned as a barrier in use of MMNP. Since the mothers were already users of MMNP who could buy them at a price, perhaps the poorest families were never included and cost as barrier was not evident.

Schedule preference was one of the significant predictors of high adherence. Mothers who preferred feeding flexibly were less likely to have high adherence. The program employed a daily feeding schedule of MMNP. In spite of that 24% of mothers in the study preferred to feed every alternate day which was the previous schedule. The mothers have not been oriented and motivated well to follow the new schedule which has affected their adherence. The SS’s role in communicating messages and motivating mothers is once again highlighted.

Side effects have traditionally been a concern with other iron supplements as reflected by studies on pregnant women [28,33,34] where adherence was negatively influenced. Nevertheless, there are also studies that concluded the other way round [35,36]. In case of MMNP, studies from Bangladesh [13,25] have reported change in color and consistency of stool as common side effects in children. In this study, only a negligible percentage (1.3%) of mothers reported negative effect (vomiting) otherwise most were positive health effects including increased appetite which was also reported by other studies [12,13,25,37]. A recent cluster randomized trail from Pakistan has shown the use of MMNP to be associated with a significant increase in diarrhea incidence (RR = 1.04, 95% CI: 1.01-1.06) [38]. In our study mothers did not specifically reported diarrhea as a side effect, however around 13% of children had diarrhea in the past 15 days. Here we should note that the period of MMNP use and period of diarrhea (past 15 days) may or may not overlap. Therefore, it would be difficult to assume that the use of MMNP may have been associated with diarrhea.

Contrary to other studies [12,25] nearly half of the mothers perceived changes in food after mixing MMNP, primarily in color. However these changes did not deter them from continuing feeding MMNP akin to results of study by Karim et al. in 2006. Median acceptability ratings of organoleptic properties were comparable to a recent study from Bangladesh which found overall acceptability of MMNP to be 5 (“Liked a lot”) on a 5 point hedonic scale [37]. A Mexican study among women also reported similar results where median ranking of organoleptics and use quality was 2 (“I liked it”) on a 5 point Likert scale. The studies have cited unwillingness of study participants to give negative answers or cultural bias of Likert scales as probable explanation of homogeneous response obtained [24] which may also be valid for our study. We also felt that mothers had difficulty understanding acceptability questions and responding on behalf of their children. Furthermore, for a starving child in a poor household who would rarely be fed a full meal, acceptability of food whether mixed with MMNP or not may not be an issue at all. A qualitative inquiry perhaps could have elicited insightful results.

Some limitations should be considered when interpreting the findings. This study calculated adherence among families who purchased the product. These families have already made an investment in the product and its use, so adherence may be very different compared to a population where MMNP are distributed for free. We need to be careful when generalizing findings from this study. But at the same time, this study will give an insight of MMNP program in Bangladesh. Previous studies in Bangladesh measured adherence in a controlled setting by counting the number of used and/or unused sachets on a weekly or monthly basis [12-14,25]. In this study empty/used sachets were not available while unused sachets could be counted only in those cases where the child was currently being fed and not in middle of a break. Further, SS registers were not up to date for us to obtain the dates when MMNP was sold. Consequently we had to rely on mother’s ability to recall dates in order to calculate adherence. Thus, there may be memory or reporting bias. In addition, respondents within a cluster/SS catchment area may not be heterogeneous in study characteristics as they may be influenced by the SS’s knowledge and skills. However given that such effects if any were minimized during sample size calculation and data analysis, we are convinced that findings represent actual situation and are representative of a rural community.

To our knowledge this is the first cross sectional study in Bangladesh that measured adherence and associated factors in a community setting. There are several implications of this study for the improvement of the BRAC MMNP program in particular as well as for other MMNP programs elsewhere that operate through community health workers. First, the role of SS in ensuring high adherence is central as they are the ones who sell, inform, motivate and monitor usage among mothers. Thus it is imperative to come up with ways to ensure that they have appropriate knowledge and skills to perform the task with high levels of motivation. It may be achieved through training and incentives.

Second, monitoring of adherence is possible only through proper record keeping of sales, usage and timing of dosage. SS registers could be used but were not up to date on MMNP sales. Alternately, a compliance card could be introduced similar to an immunization card to keep track of children under the program. Third, information such as change in feeding schedule should be communicated well and the mothers should be motivated to adhere to such changes.

## Conclusions

SS are the key to improving adherence through regular visits to households of MMNP users. However, expanding coverage beyond the vicinity of the SS’s household is a challenge. In the light of evidence from this study it is difficult to interpret whether an adherence of 70% is acceptable for a nutritional intervention within a program setup. The fact that the mothers did not mention any barriers to MMNP usage is encouraging. Additionally acceptability and perceived negative effects were not a big concern. Nonetheless, registers should be up to date. An updated register will be useful in tracking mothers who have bought MMNP and ensuring adherence. Timely and regular supply could be a problem but it was not investigated in this study.

The implementation of these results will help the second phase of the BRAC MMNP larger program in designing and improving adherence among users of MMNP. In the future, biological samples may be collected concurrently with cross sectional measurement of adherence. There after hemoglobin levels in blood may be determined at different levels of adherence. Thus it would help establish the appropriate level of adherence that is needed to maintain adequate blood hemoglobin levels. Studies should also focus on the nature of interaction between SS and mothers to gain better understanding of factors that come in to play during SS visits. Additionally perception of families whose children have low adherence should be studied.

## Endnote

^a^Fever (Mother’s Perception); Defecate 3 or more times in a day; Watery stool; Mucus or blood with stool; Stomachache; Nausea; Vomiting; Turn in to blue (any part of the body); Itching in the body or head; Toothache; Always cough; Running nose; Wheezing
